# Qatar Diabetes Mobile Application Trial (QDMAT): an open-label randomised controlled trial to examine the impact of using a mobile application to improve diabetes care in type 2 diabetes mellitus—a study protocol

**DOI:** 10.1186/s13063-022-06334-5

**Published:** 2022-06-16

**Authors:** Noor Suleiman, Meis Alkasem, Zaina Al Amer, Obada Salameh, Noora Al-Thani, Mohammad Khair Hamad, Khaled Baagar, Ibrahem Abdalhakam, Manal Othman, Ragae Dughmosh, Dabia Al-Mohanadi, Ali Al Sanousi, Mohammed Bashir, Odette Chagoury, Shahrad Taheri, Abdul-Badi Abou-Samra

**Affiliations:** 1 Qatar Metabolic Institute, Hamad Medical Corporation, Doha, Qatar; 2 Department of Diabetes and Endocrinology, Hamad Medical Corporation, Doha, Qatar; 3 Department of Medicine, Weill Cornell Medicine – Qatar, Doha, Qatar; 4 Clinical Information Systems Department, Hamad Medical Corporation, Doha, Qatar; 5 Department of Medicine, Weill Cornell Medicine – New York, New York, USA

**Keywords:** Type 2 diabetes mellitus, Self-management, Mobile health, Lifestyle change, Clinical trial

## Abstract

**Background:**

Mobile health (mHealth) is increasingly advocated for diabetes management. It is unclear if mobile applications are effective in improving glycaemic control, clinical outcomes, quality of life and overall patient satisfaction in patients with type 2 diabetes (T2DM). A new mobile application was specifically built for people with T2DM with the help of the local expertise. The objective of the study was to evaluate the effectiveness of the mobile app.

**Methods:**

The planned study is an ongoing open-label randomised controlled trial in which adults living with T2DM treated with insulin will be randomised 1:1 to the use of this diabetes application versus current standard care. The primary outcome will be the difference in mean HbA1c from baseline to 6 months. Other outcome measures include anthropometric measures, hypoglycaemic events, medication adjustments, number of clinical interactions and missed appointments and patient perceptions of their disease and diabetes self-management. The study will randomise 180 subjects for assessment of the primary outcome.

**Discussion:**

We hypothesise that the diabetes-specific mobile application will improve glycaemic control, increase patient empowerment for self-management of diabetes and improve interaction between patients and healthcare providers. If the Qatar Diabetes Mobile Application Trial (QDMAT) demonstrates this, it will inform clinical services for the future self-management of T2DM.

**Trial registration:**

ClinicalTrials.gov Identifier: NCT03998267. Registered on 26 June 2019

## Administrative information

Note: the numbers in curly brackets in this protocol refer to SPIRIT checklist item numbers. The order of the items has been modified to group similar items (see http://www.equator-network.org/reporting-guidelines/spirit-2727-statement-defining-standard-protocol-items-for-clinical-trials/).

**Table:** 

Title {1}	Qatar Diabetes Mobile Application Trial (QDMAT): A randomised controlled trial protocol publication
Trial registration {2a and 2b}	ClinicalTrials.gov NCT03998267
Protocol version {3}	Version 11 28.04.2021
Funding {4}	Medical Research Center at Hamad Medical Corporation, Doha, Qatar
Author details {5a}	1. Qatar Metabolic Institute, Hamad Medical Corporation, Doha, Qatar 2. Department of Diabetes and Endocrinology, Hamad Medical Corporation, Doha, Qatar 3. Department of Medicine, Weill Cornell Medicine – Qatar, Doha, Qatar 4. Clinical Information Systems Department, Hamad Medical Corporation, Doha, Qatar 5. Department of Medicine, Weill Cornell Medicine – New York, New York, USA
Name and contact information for the trial sponsor {5b}	Dr Noor Suleiman, Qatar Metabolic Institute, Hamad Medical Corporation, Doha, Qatar
Role of sponsor {5c}	The sponsor institution (HMC) played will take no part in study design, collection, management, analysis, and interpretation of data, writing of the report, and the decision to submit the report for publication.

## Introduction

### Background and rationale {6a}

Type 2 diabetes Mellitus (T2DM) is a chronic disease associated with serious micro- and macro-vascular complications that reduce quality of life and longevity with adverse social and economic consequences [1]. T2DM prevalence is increasing globally but at a more rapid pace in the Middle East and North Africa (MENA) region, and particularly in Arab countries in the Gulf Cooperation Council [2, 3]. T2DM management is focused around glycaemic, blood pressure and lipid control [4]. However, even with well-established and enhanced clinical services, achieving appropriate clinical and biochemical targets to prevent, diminish and reverse diabetes complications, is challenging [5]. This is because T2DM management is a complex process requiring integrated clinical care and self-management.

Given the brevity of clinical encounters because of lack of capacity to cater for the increasing number of patients with T2DM, self-management is vital to T2DM care [6]. T2DM self-management requires comprehensive patient education on lifestyle, behavioural change, medication management and adherence and dealing with specific circumstances such as acute illness (“sick day” rules), supported by the clinical diabetes management team. Self-monitoring generally includes blood glucose measurements for people with T2DM treated with insulin but may also include blood pressure, body weight, physical activity and dietary intake measures. As T2DM progresses, there is a need for treatment intensification and the introduction of insulin, which is associated with hypoglycaemia and undesirable weight gain [4, 7]. People with T2DM treated with insulin require more frequent support and healthcare contact especially when insulin is initiated or insulin dose is altered. Providing this through the standard clinic setting is challenging.

T2DM management is increasingly supported by mobile health (mHealth) technologies [8–10] allowing self-monitoring and peer interaction as well as remote monitoring, education, coaching [11] and support by healthcare professionals [12–14]. Furthermore, mHealth allows more convenient remote interaction between the patient and healthcare providers. Thus, mHealth approaches could not only improve T2DM self-management but also allow a more efficient and cost-effective platform for managing the growing number of patients.

Several types of smartphone apps exist for T2DM self-management [15]. The characteristics of these apps, including increased accessibility to educational resources and self-management strategies, more frequent physical and emotional symptom tracking and increased access to peer support, are strengths in benefiting patients' self-management. Based on systematic review, mobile phone-based interventions with clinical feedback improve glycaemic control (HBA1c) compared to standard care or other non-mHealth approaches by as much as 0.8% (9 mmol/mol) for people with T2DM and 0.3% (3 mmol/mol) for people with type 1 DM (T1DM), at least in the short-term (12 months) [13]. The effectiveness of mHealth apps is likely to be influenced by local culture and population mix. There are few studies of mHealth using mobile apps in Arab countries. mHealth interventions are likely to be well-received in Arab countries where the majority of patients with DM are young [16, 17] and likely to be more proficient with mobile app use. Also, digital platforms are increasingly used to seek health information by patients, demonstrating a wish to embrace digital technology for healthcare [18]. In a pilot study of 20 patients with T2DM in Saudi Arabia, there was a reduction in HBA1c from 8.8% (7 mmol/mol) to 7.9% (63 mmol/mol) in the mHealth intervention group compared to a change from 8.6% (70 mmol/mol) to 8.7% (72 mmol/mol) in the control group [19]. The mean diabetes knowledge for the intervention group rose significantly from 46.2 to 61.1% in 6 months using an adapted Diabetes Knowledge questionnaire. The control group also showed slight increase from 43.7 to 46.1% [19].

Recently, the Droobi app (https://en.droobihealth.com/) was co-created by healthcare professionals, patients and computer scientists as a platform for DM management for the Arabic speaking population catering for shared language and sociocultural factors [20]. The Droobi app provides an avenue for more frequent and personalised contact amongst patients, diabetes educators, dietitians and physicians. Rather than focusing on a single functionality as found in many diabetes related mobile apps [15], the Droobi app has three key functionalities: (i) self-management through recording of key measures such as capillary glucose readings, diet, and activity; (ii) education platform for lifestyle support and (iii) real-time interaction with healthcare professionals (physicians, diabetes educators, and dietitians). The aim of the Qatar Diabetes Mobile Application Trial (QDMAT) is to examine the impact of including the Droobi app in diabetes care on diabetes outcomes compared to standard diabetes care. The clinical trial is conducted in Qatar which benefits from a multinational Arab population with a high prevalence of chronic diseases including diabetes [16, 21].

### Objectives {7}

QDMAT will examine the hypothesis that the use of the Droobi diabetes management mobile app platform results in better glycaemic control in patients with T2DM compared to standard care. Secondary outcomes of interest include other key diabetes outcome measures (e.g. blood pressure) and diabetes self-care.

### Trial design {8}

QDMAT is an ongoing open-label parallel group randomised clinical trial. Participants with T2DM who are treated with insulin will be randomised with a 1:1 ratio into an intervention arm and standard care arm. Participants in the intervention arm will receive care through the Droobi mobile app in addition to their standard care. Participants in the control group will receive the usual DM care based on national and international DM care guidelines [22, 23].

A diabetes population comparator group will be used to examine the natural progression of diabetes and its treatment outside the randomised controlled trial. This population will contextualise and determine the effectiveness of the trial arms compared to a non-randomised population.

## Methods: participants, interventions and outcomes

### Study setting {9}

QDMAT is conducted in a secondary care diabetes care setting at Hamad Medical Corporation (HMC), the largest national public medical care provider in Qatar.

### Eligibility criteria {10}

#### Inclusion criteria

The following will be included in QDMAT:

- Adults with T2DM (based on clinical records);
- 18–60 years of age;
- Uncontrolled DM with HBA1c ≥ 8.5% (69 mmol/mol);
- Insulin treated T2DM;
- Arabic or English language speaking and able to read and write in one of these languages;
- Possession of a smart mobile phone and willing to use a smart phone app;
- Able to communicate via chat with the mobile app team through the app as evidenced by at least weekly use of any of the social media apps;
- Willing to utilise a mobile application for DM management.

#### Exclusion criteria

The following will be excluded in QDMAT—those with:

- A recent history (3 months) of stroke or myocardial infarction;
- Proliferative diabetic retinopathy;
- Any acute illness in the past 2 weeks;
- Any planned absence for more than 3 months;
- End-stage renal disease requiring renal replacement therapy;
- Hypoglycaemia unawareness;
- More than one episode of severe hypoglycaemia in the previous 6 months;
- Female gender who are planning for pregnancy in the coming 6 months;
- Visual impairment preventing use of the app.

The inclusion criteria aim at involving patients with type 2 diabetes who are uncontrolled on insulin therapy who need closer monitoring and management. Having a phone with the technical ability to support the app in addition to the participant’s willingness to use the technology are key for testing the intervention.

The key exclusion criteria aim at reducing the risk of severe hypoglycaemia and potential deterioration of diabetes complications with short-term glycaemic improvement (e.g. proliferative retinopathy may be worsened by rapid improvements in glycaemic control). An acute illness may result in acute blood glucose fluctuations. Recent stroke or myocardial infarction may render the participant at risk of hypoglycaemia.

### Who will take informed consent? {26a}

All written informed consent will be taken by trained clinical research personnel listed on the trial delegation log. A two-stepped process was adopted for the study to cater for potential participants without recent biochemical investigations and to aid rapid study recruitment. Potential participants will be consented initially to screen for study eligibility with up-to-date biochemical measures. If eligible for the study, participants will be consented after more detailed explanation of the research and randomised into the randomised clinical trial.

### Additional consent provisions for collection and use of participant data and biological specimens {26b}

No biological specimens for research purposes will be collected as part of the study.

## Interventions

### Explanation for the choice of comparators {6b}

The comparator group with be standard diabetes care following national and international guidelines [22, 23]. Since in clinical trials, the comparator group often benefits from improved outcomes through trial participation, as part of the study, an observational matched diabetes population comparator group will also be examined using data from electronic medical records. This will contextualise and determine the effectiveness of the trial arms compared to a non-randomised population.

### Intervention description {11a}

#### The Droobi app

**Fig. 1:**
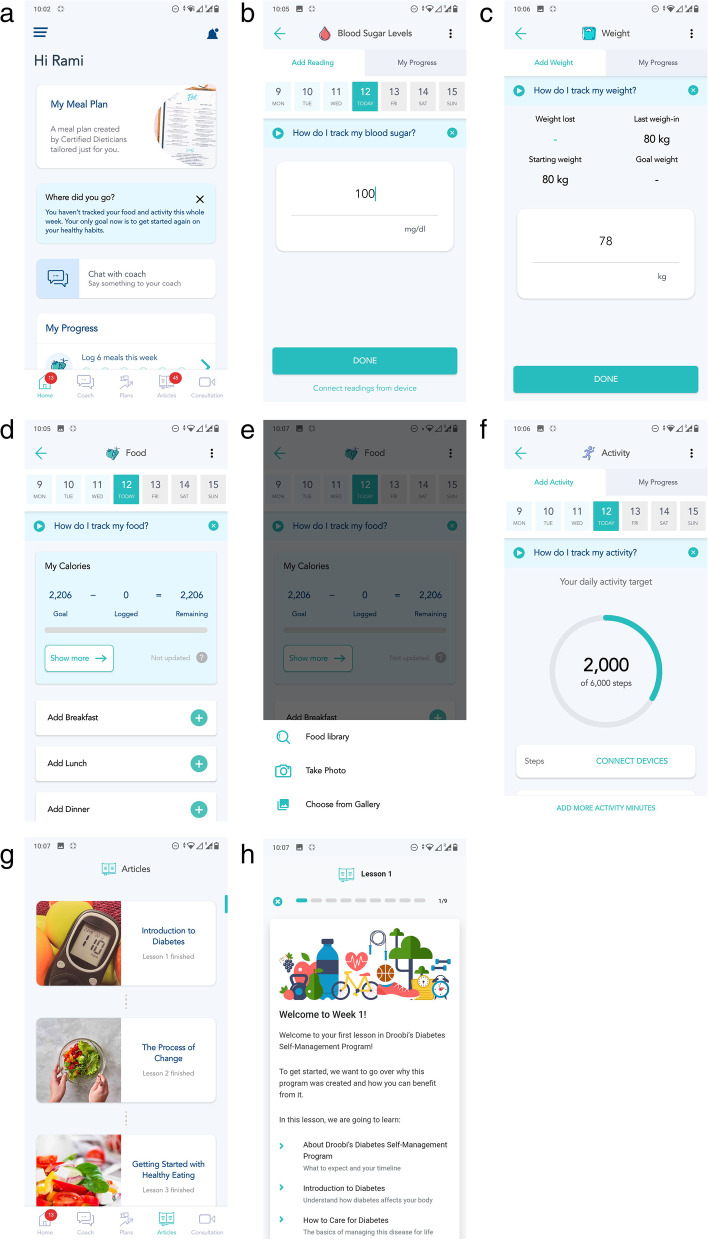
The Droobi app. **a** Home/landing page. **b** Blood glucose level entry. **c** Body weight entry. **d** Food portions entry. **e** Food photo entry. **f** Activity entry. **g** Toolbox educational curriculum. **h** Lesson 1 from the curriculum

The Droobi app is provided to participants at no cost. The app is currently available in both Arabic and English languages. It allows participants to enter self-measured blood glucose readings, weight, food entries, communicate with their physician, diabetes educator and dietitian through a chat function, together with reviewing their medication plan and the educational curriculum on diabetes. Figure 1 shows the app’s patient interface. Participants are alerted when they do not enter their blood glucose readings and when their medication plan or blood glucose monitoring frequency has been adjusted.

**Fig. 2:**
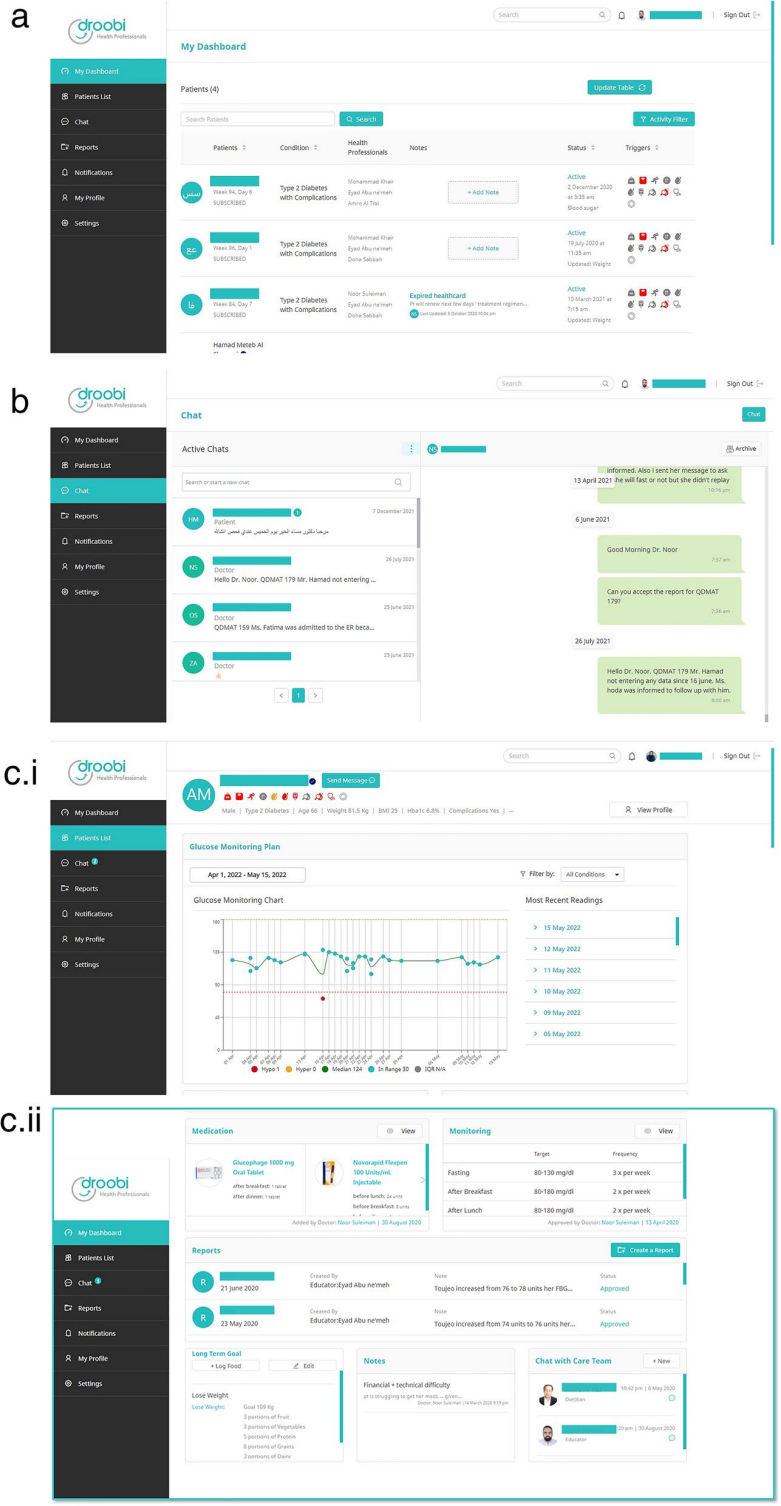
The Droobi app healthcare provider portal. **a** Portal landing page-dashboard. **b** Chat function amongst healthcare team. **c** Report created by Droobi upon request of educator for physician approval: (i) expanded report view showing glucose monitoring chart and ability to review previous blood glucose readings in chart and table format; (ii) expanded report view showing medication and blood glucose monitoring plan together with diet goals, notes for the team and the assigned healthcare team

Healthcare providers access through a Droobi portal (Fig. 2). Here, they can review the list of participants and can have an overview of active participants and any triggers highlighted (i.e. low or high blood glucose entries) in order to prioritise care. Also, individual participants blood glucose readings and food entries may be reviewed and their medications and frequency of monitoring adjusted as needed together during chats with the assigned healthcare providers. Educators send a report for the assigned physician in order to adjust participant’s medication plan (Fig. 2).

The Droobi app has been designed to be simple to use, mimicking existing social media tools. Participant training for use of the app will take place with the educator and technical support team during the initial participant set up phase. The following steps are taken:

i. Educate/train participant on app usage;
ii. Participants will be subscribed to the app and their profile on the app will be created;
iii. Participants will log in their blood glucose readings and communicate with the mobile app team (educators and physician) via the app;
iv. Participants will be placed on a diet and lifestyle plan as agreed upon by the participant and healthcare provider team, best suited towards the participant’s needs;
v. Throughout the study, participants will receive notifications and advice on how to follow diet and lifestyle changes;
vi. Throughout the study; participant interaction and app usage will be tracked.

During the trial, there will be a one-to-one training session with the participant, displaying the mobile application and its key features in the preferred language. Live demonstration on the participant’s phone will be performed, and the participant is instructed to demonstrate application use in the presence of the team to ensure that they are able to use the application easily. The team ensures the participant’s training checklist is completed. Also, a printed manual will be provided, which will also include advice for minimising patient risk (e.g. no app is substitute for medical advice, especially during medical emergencies, and how to use the app and protect privacy). Similar training will be conducted for the educators and physicians involved in the intervention arm.

All training will be carried out by the app development team involved in the study, together with the investigators facilitating interaction. For any technical questions or concerns, all participants (both study participants and healthcare professionals) will have available user support platform during the study duration in addition to a Droobi technical hotline number.

#### Standard care

At baseline, participants will be seen by the dietitian and diabetes educators in the diabetes clinic as part of standard care. The educators contact telephone number, and a diabetes hotline number will be provided to all as standard. The diabetes hotline number is a service provided to patients with diabetes to help communicate with the diabetes educators with questions relating to their diabetes management, medication adjustment such as dose titrations, etc. Appointments thereafter with the educator and/or dietitian will be decided and scheduled according to individual need, with a planned minimum visit every 3 months during the study period.

#### The healthcare provider team

Mobile app intervention team: This team consists of healthcare providers comprising of physician, diabetes educators and dietitians who provide feedback and coaching to participants through the app using all clinical and self-entered information available within the app.

Standard care team: This team consists of healthcare providers comprising of physician, diabetes educators and dietitians providing standard diabetes care based on national and international diabetes guidelines.

Insulin doses were adjusted based on multi-professional assessment to avoid any risks associated with hypoglycaemia.

For the observational comparator population, patients records will be identified via electronic medical records to match inclusion/exclusion criteria of QDMAT participants.

### Criteria for discontinuing or modifying allocated interventions {11b}

Participants may withdraw from the study at any time without their treatment or access to services being affected. Investigators may withdraw participants if they develop a condition that excludes them from the study, e.g. cancer.

### Strategies to improve adherence to interventions {11c}

Participants are contacted in a timely manner to ensure they are reminded of their laboratory investigations and physician's appointments together with the dietitian and educator appointments. Also, the intervention group receive reminders from the healthcare providers on the mobile app. Any difficulties in using the app will be addressed by the healthcare provider and app technical team.

### Relevant concomitant care permitted or prohibited during the trial {11d}

All participants will have access to any required medical care.

### Provisions for post-trial care {30}

Once the intervention completed, the mobile app for the intervention group will be deactivated, and they will be returned to standard diabetes care. In case of injury, care will be provided at HMC without cost, but any outside care will be at the participant’s expense.

### Outcomes {12}

#### Primary outcome

The primary outcome is the difference in mean HbA1c (change from baseline) between the intervention arm and the standard care arm at 6 months based on available resource.

#### Secondary outcomes

Secondary outcomes include differences in:

- Perceptions of diabetes self-management as assessed by diabetes self-management questionnaire (DSM-Q) [24]; a self-completed questionnaire including 16 items assessing selfcare activities associated with glycaemic control at 0 and 6 months;
- Attitudes towards disease assessed using the diabetes distress scale (DDS) [25]; a 17-item participant-completed questionnaire assessing psychosocial distress in diabetes at 0 and 6 months;
- Number of recommended insulin dose adjustments;
- Number of reported hypoglycaemic events;
- The time required to reach normoglycaemia;
- The number of clinical interactions;
- The proportion of missed clinical appointment;
- In anthropometric measures (body weight; body mass index [BMI]), blood pressure and lipid profile;
- Key outcome measures beyond 6 months with follow-up (up to 5 years).

#### Exploratory outcomes and additional analyses

Exploratory outcomes and additional analyses include:

- Further analysis of patient characteristics in terms of responders and non-responders to the mobile intervention;
- Reduction in hospital admissions;
- Comparison of groups to the observational cohort will be made.

Adverse events: adverse events will be recorded throughout the trial.

### Participant timeline {13}

**Fig. 3:**
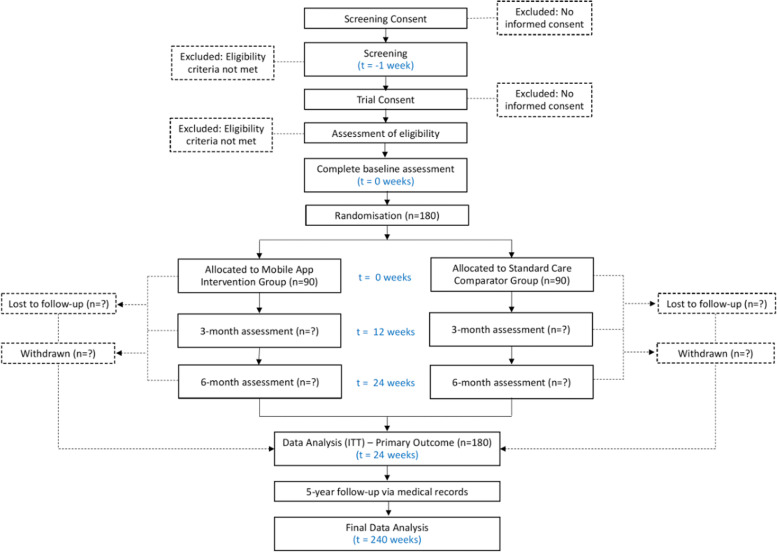
Flow of participants

Table 1 shows the schedule of visits and assessments, and Fig. 3 shows the flow of participants.

**Table 1:**
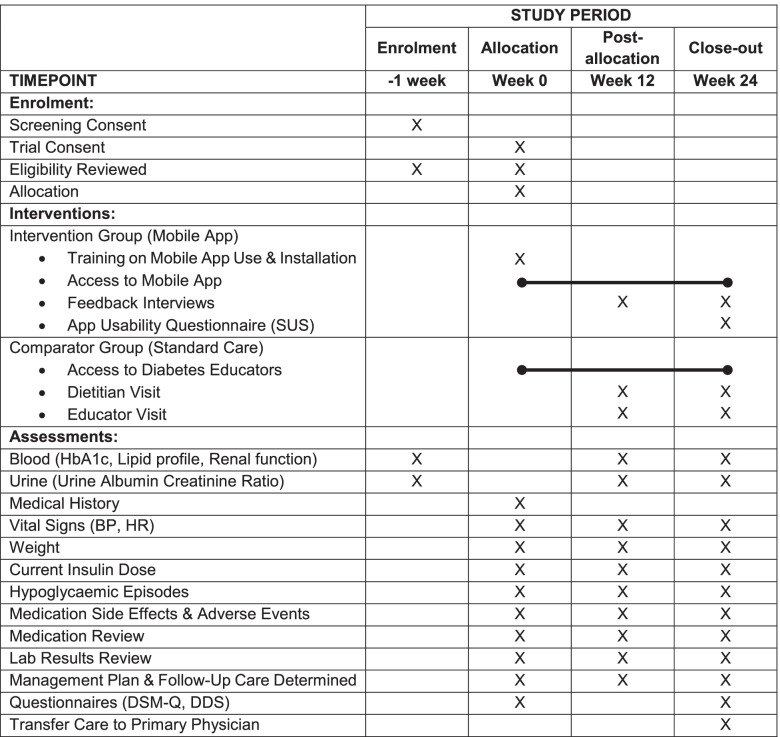
Schedule of enrolment, intervention, study visits and assessments for both study groups

*SUS* System Usability Scale, *BP* blood pressure, *HR* heart rate, *DSM-Q* Diabetes Self-Management Questionnaire, *DDS* Diabetes Distress Scale

Expected duration: the intervention will be for 6 months with further follow-up to examine distal clinical outcomes of interest. Participants consent to follow-up of up to 5 years.

Participants must complete at least visit 0 and 6 months to be considered as study completers.

### Sample size {14}

The initial sample size was based on a clinically relevant difference in HbA1c of 0.5% (5.5 mmol/mol) with SD 1% (11 mmol/mol) between the intervention and the standard care arms [26]. To achieve an 80% power and a significance level of < 0.05, 64 participants would be required in each arm. Assuming a 40% loss to follow-up, 90 participants were planned to be recruited in each arm. However, review of baseline characteristics of recruited participants by the trial steering group identified a higher SD of 1.7% (19 mmol/mol) giving 182 participants per arm, with a total recruitment of 608 participants to include a 40% dropout. Therefore, it was decided to extend recruitment beyond the initially planned number to 608 participants. Sample size calculations were made using STATA 15 MP. For the population cohort comparator group, we aim to select a sample 200 patients identified through the available diabetes registries to examine the impact of trial arms compared to a non-randomised group.

### Recruitment {15}

Recruitment will be from diabetes clinics at Hamad Medical corporation, pharmacy lists (insulin treatment), and diabetes registries. For the cohort observational group, identification will be via existing electronic medical records. Recruitment will also make use of approved social media announcements. Potential participants will be provided with the study information sheet prior to consent.

## Assignment of interventions: allocation

### Sequence generation {16a}

Study participants shall be randomised to one of the study arms using minimisation on age (18–40 years; 41–60 years), gender, duration of diabetes (≤ 5 years; > 5 years), and medications (oral + basal insulin; oral + basal/bolus insulin). Biphasic insulin intake was assigned to the basal/bolus group.

### Concealment mechanism {16b}

Randomisation will be done through a web-based randomisation system (www.sealedenvelope.com) accessed by study research personnel.

### Implementation {16c}

The allocation sequence was provided by the online randomisation service. The allocation is generated once participant eligibility criteria are entered into the platform by clinical research coordinators blinded to any allocation.

## Assignment of interventions: blinding

### Who will be blinded {17a}

Due to the nature of the intervention, participants and the research team delivering the intervention will not be blinded to the treatment received. Those involved in the data analyses and statistics will be blinded to the group allocation.

### Procedure for unblinding if needed {17b}

There are no required processes as the trial is open label.

## Data collection and management

### Plans for assessment and collection of outcomes {18a}

The data collection team consists of research assistants who will collect key outcome. A study specific case report form (CRF) has been designed for collection of all related data required to analyse the study outcomes. The data is mostly captured on electronic medical records and copied into the CRFs on a periodic basis by the research assistants. The study investigators regularly scrutinise the data for any inconsistencies or missing information. The forms are maintained in the patient files and an electronic copy is fed into the study database for future analyses.

### Plans to promote participant retention and complete follow-up {18b}

A robust plan aims to encourage app use and all technical problems are resolved expediently by the technical team. Regular contact with trial participants will ensure retention.

### Data management {19}

All personal data will be coded, and all patients enrolled into the study will be given a unique and confidential participant ID only accessible to the research team. The unique IDs and data collected will be stored in the database in an encrypted format. The database that contains personal information of the patient will be kept separate from the database containing their health information. The only connection between the databases will be the unique Identifier which again will be encrypted throughout the study and during data transmission. The data entered by study participants will be directly sent over a secure line to the database for storage. There will be no third party involved in the transmission of data. Coded data without the allocation will be provided for data analysis.

### Confidentiality {27}

All identifiable data will be kept securely in locked cabinets in a locked room. Electronic data will be kept encrypted on secure servers with specific log access by research personnel. The authorised personnel will only be able to access the minimum necessary information needed to perform job functions.

### Plans for collection, laboratory evaluation and storage of biological specimens for genetic or molecular analysis in this trial/future use {33}

Blood samples collected during the study shall be used for the purpose of this trial alone. Any leftover samples will be discarded at the end of the study.

## Statistical methods

### Statistical methods for primary and secondary outcomes {20a}

Descriptive statistics will be used to summarise and determine the sample characteristics and distribution of patients’ data. The normally distributed data and results will be reported with mean and standard deviation (SD) with corresponding 95% confidence interval (CI); the remaining results will be reported with median and inter-quartile range (IQR). Categorical data will be summarised using frequencies and proportions. Associations between two or more qualitative data variables will be assessed using chi-square (*χ*^2^) test or Fisher exact test, as appropriate. Quantitative data between the two independent groups will be analysed using unpaired *t* or Mann-Whitney *U* test as appropriate.

The analysis will follow the intention to treat principle with last observation carried forward. Difference in mean change from baseline HBA1c between the intervention arm and the standard care arm at 3 and 6 months will be analysed using repeated measure ANOVA method, linear regression and analysis of covariance (ANCOVA) methods as appropriate. Sensitivity analysis will be conducted using other approaches based on data available. Comparisons will be conducted between trial group and the observational cohort using the approaches described above.

### Interim analyses {21b}

There are no planned interim analyses.

### Methods for additional analyses (e.g. subgroup analyses) {20b}

Exploratory analyses will examine differences between app responders and non-responders using the analyses described above.

### Methods in analysis to handle protocol non-adherence and any statistical methods to handle missing data {20c}

The data related to participants who voluntarily withdraw from the study or lost to follow-up (non-adherence) shall be analysed with an “intention to treat” analysis.

### Plans to give access to the full protocol, participant level-data and statistical code {31c}

The full protocol will be available for review. All other data will be available from the study chief investigator upon reasonable request.

## Oversight and monitoring

### Composition of the coordinating centre and trial steering committee {5d}

The study is single-centre and the steering committee comprises of the study investigators.

### Composition of the data monitoring committee, its role and reporting structure {21a}

Given the open-label of the study and no expected unexpected events, data and safety monitoring will be conducted by the investigator team.

### Adverse event reporting and harms {22}

All adverse events are reported to the institutional review board (IRB) with 24 h for serious adverse events and at continuing review for non-serious events.

### Frequency and plans for auditing trial conduct {23}

The study will be submitted to the HMC IRB for review and approval. Annual continuing review will be conducted by the IRB. The HMC Medical Research Center (MRC) will conduct monitoring and audit the study; this supplements internal monitoring by the investigative team.

### Plans for communicating important protocol amendments to relevant parties (e.g. trial participants, ethical committees) {25}

Any changes to the protocol will be submitted to the HMC IRB for approval prior to implementation. All investigators and research personnel will be notified of any protocol changes. Any changes that may impact participant care or decision to participate will be communicated to participants and they will be reconsented using a newly edited approved consent form.

### Dissemination plans {31a}

Results from the current study will be summarised and reported annually to the Diabetes and Endocrinology Division at HMC, Qatar. Findings from the study will also be disseminated to primary healthcare in Qatar through joint webinars. Further dissemination will occur via the National Diabetes Strategy Committee (members: ST and AB-S) at the Ministry of Public Health, Qatar. Further dissemination will occur through local, regional and international clinical and scientific meetings and through publications in general and disease-specific journals. Important findings will be shared with the public at large through media outlets.

## Discussion

QDMAT is the first randomised clinical trial in Qatar aiming to test whether the use of a mobile app to support medical and self-management of insulin-treated T2DM results in improved glycaemic outcomes. Little is known about conducting clinical trials of mHealth interventions in Qatar, and the progress of the trial will inform future similar interventions. Anticipated challenges to the trial conduct include subject recruitment and retention. While the population of Qatar report willingness to participate in clinical studies and those who have participated in previous studies report a positive experience, there are several barriers to study participation [21]. These barriers are common to studies outside Qatar and include time commitment for study participation and lack of awareness of clinical research. This may not be a barrier in QDMAT since the use of the mobile app allows participants to manage diabetes remotely. Also, all assessments are conducted simultaneously with planned clinical visits. Furthermore, the population with diabetes in Qatar are younger than western populations and use technology frequently for health purposes [17, 18]. Recruitment into clinical research studies has benefitted greatly from the introduction of electronic medical records across Qatar (in both primary and secondary care) and diabetes registries. This allows rapid identification of patients for targeted clinical care as well as for research participation [27]. Qatar has a diverse population including a large expatriate population which can affect participant retention and long-term follow-up. Longer residence in Qatar is associated with greater willingness to enrol in clinical research and greater probability of participant retention [21]. The 6-month duration of QDMAT should ensure that the majority of study participants remain within the country.

## Trial status

The trial is ongoing with first subject randomised on 22 August 2019

Protocol version: version 9 dated 10 January 2021

Date recruitment began: 22 August 2019

Approximate date when recruitment will be completed: 01 May 2021

## Abbreviations

App: Mobile application
DM: Diabetes mellitus
HbA1c: Haemoglobin A1c (glycated haemoglobin)
HMC: Hamad Medical Corporation
IRB: Institutional Review Board
MRC: Medical Research Center
T2DM: Type 2 diabetes
T1DM: Type 1 diabetes
